# A cross-sectional study of differences in 6-min walk distance in healthy adults residing at high altitude versus sea level

**DOI:** 10.1186/2046-7648-3-3

**Published:** 2014-02-01

**Authors:** Deirdre Caffrey, J Jaime Miranda, Robert H Gilman, Victor G Davila-Roman, Lilia Cabrera, Russell Dowling, Talia Stewart, Antonio Bernabe-Ortiz, Robert Wise, Fabiola Leon-Velarde, William Checkley

**Affiliations:** 1 Division of Pulmonary and Critical Care, School of Medicine, Johns Hopkins University, 1800 Orleans St, Suite 9121, Baltimore, MD 21205, USA; 2 CRONICAS Center of Excellence in Chronic Diseases, Universidad Peruana Cayetano Heredia, Lima 31, Peru; 3 Departamento de Medicina, Escuela de Medicina, Universidad Peruana Cayetano Heredia, Lima 31, Peru; 4 Program in Global Disease Epidemiology and Control, Department of International Health, Bloomberg School of Public Health, Johns Hopkins University, Baltimore 21205, USA; 5 A.B. PRISMA, Lima 32, Peru; 6 Cardiovascular Imaging and Clinical Research Core Laboratory, Cardiovascular Division, Washington University School of Medicine, St. Louis 63110, USA; 7 Departamento de Ciencias Biológicas y Fisiológicas, Laboratorio de Adaptación a la Altura, Universidad Peruana Cayetano Heredia, Lima 31, Peru

**Keywords:** Six-minute walk test, High altitude adaptation, Hypoxia, Functional capacity

## Abstract

**Background:**

We sought to determine if adult residents living at high altitude have developed sufficient adaptation to a hypoxic environment to match the functional capacity of a similar population at sea level. To test this hypothesis, we compared the 6-min walk test distance (6MWD) in 334 residents living at sea level vs. at high altitude.

**Methods:**

We enrolled 168 healthy adults aged ≥35 years residing at sea level in Lima and 166 individuals residing at 3,825 m above sea level in Puno, Peru. Participants completed a 6-min walk test, answered a sociodemographics and clinical questionnaire, underwent spirometry, and a blood test.

**Results:**

Average age was 54.0 vs. 53.8 years, 48% vs. 43% were male, average height was 155 vs. 158 cm, average blood oxygen saturation was 98% vs. 90%, and average resting heart rate was 67 vs. 72 beats/min in Lima vs. Puno. In multivariable regression, participants in Puno walked 47.6 m less (95% CI -81.7 to -13.6 m; *p* < 0.01) than those in Lima. Other variables besides age and height that were associated with 6MWD include change in heart rate (4.0 m per beats/min increase above resting heart rate; *p* < 0.001) and percent body fat (-1.4 m per % increase; *p* = 0.02).

**Conclusions:**

The 6-min walk test predicted a lowered functional capacity among Andean high altitude vs. sea level natives at their altitude of residence, which could be explained by an incomplete adaptation or a protective mechanism favoring neuro- and cardioprotection over psychomotor activity.

## Background

Several studies have examined differences in exercise capacity between acclimated, high altitude residents, and non-acclimated controls [1-3]. Whether due to genetic or developmental adaptations to hypoxic environments, high altitude natives have higher exercise performance at altitude when compared to lowlanders at high altitude [4]. Differences between highlanders and lowlanders have been studied by measures such as maximal oxygen consumption (VO_2 max_), pulmonary ventilation, pulmonary gas exchange efficiency, muscle response, arterial oxygen saturation, and lactate buffering capacity [5].

Individuals non-acclimated to altitude have a decrease in VO_2 max_ upon assent to high altitude [5,6]. In contrast, VO_2 max_ is greater in highland dwellers of similar age, sex, and body proportions [2,7-9], suggesting that they may be better adapted to live in hypoxic environments than lowlanders. The present study uses the 6-min walk test (6MWT) to compare functional capacity between high altitude residents and lowlanders in their respective environments.

The 6MWT has a variety of uses in clinical settings to test a patient's response to disease or medical interventions, their functional capacity, and their predicted morbidity and mortality [10-16]. This test was developed in 1963 to evaluate people with respiratory or heart diseases, including prognosis and functional capacity [17]. Due to the integrated systems of the body that are required during walking, the 6MWT provides information about how the cardiovascular and pulmonary systems function together. The exertion level achieved during the 6MWT is chosen by the participant and is usually less than their maximal exertion level [12]. However, activities of daily living are similarly performed at exertion levels chosen by the individual; therefore, the 6MWT is a good indicator of an individual's ability to perform activities of daily living [18].

Results from the 6MWT related to ascent to altitude have been used as a measure of changes in functional capacity among lowlanders at various altitudes, as well as a method to predict who will be affected by acute altitude sickness [19,20]. However, the 6MWT has not been used as a method to compare functional capacities of high and low altitude populations. By testing participants at their residing altitudes, this study intends to determine whether long-term high altitude residents have adapted to their environments to achieve the same functional capacity as sea level residents. Thus, our primary objective was to investigate the functional capacity of a sea level and high altitude population by means of the 6MWT.

## Methods

### Study setting

The study population consisted of adults ≥35 years of age living in the cities of Lima and Puno. Lima is the highly urbanized capital of Peru located at sea level and with a population of more than ten million. We conducted our study in Pampas de San Juan de Miraflores, a peri-urban shanty town located 25 km south of the city center. Puno is an Andean City located at 3,825 m above sea level and with a population of approximately 150,000. The average temperature was 21.6°C in Lima and 16.1°C in Puno. The study protocol was approved by the Institutional Review Boards of the Johns Hopkins Bloomberg School of Public Health in Baltimore, USA and A.B. PRISMA in Lima, Peru. All participants provided verbal informed consent after our research team read the entire informed consent document to them and any questions were answered.

### Study design

This is an ancillary study of a larger ongoing cohort study conducted in Lima and Puno. In preparation for study activities for this parent cohort study, we first conducted a door-to-door household census of the study areas from which an age-, sex- and site-stratified population-based cohort of approximately 1,000 participants per site was derived [21]. At baseline, participants responded to a face-to-face questionnaire regarding sociodemographics and medical history. Field workers measured weight, height, bioelectrical impedance, blood pressure, and spirometry before and after bronchodilators, and obtained a blood sample for analysis of cardiovascular and pulmonary biomarkers. We measured bioelectrical impedance using the TBF-300A body composition analyzer (TANITA Corporation, Itabashi-ku, Tokyo, Japan) to estimate lean mass, percent body fat, and water weight. Participants were asked to provide venous blood sample after 8 to 11 h of fasting. Blood was obtained by trained phlebotomists in the sitting position and using universal precautions. Plasma glucose was measured using an enzymatic colorimetric method (GOD-PAP; Modular P-E/Roche- Cobas, Grenzach-Whylen, Germany), serum insulin using electrochemiluminescence (Modular P-E/Roche-Cobas), hs-C reactive protein using Latex (Tina-quant CRP-HS Roche/Hitachi analyzer, Indianapolis, IN, USA), and hemoglobin A1C using high-performance liquid chromatography (D10, BioRad, Munich, Germany). We measured lung function using the Easy-On-PC spirometer (ndd, Zurich, Switzerland) following standard guidelines [22]. All patients underwent bronchodilator-response testing. We administered two puffs from a salbutamol inhaler (100 mcg/puff) via a spacer and repeated spirometry 10 to 15 min later.

We then invited a random subset of 400 participants (200 in Lima and 200 in Puno) from the parent cohort study to participate in this ancillary study. We defined healthy participants as those who did not have: a physical disability that impaired walking; self-reported diagnosis of heart failure, diabetes, asthma, COPD, active or a history of pulmonary tuberculosis; prior pulmonary or thoracic surgery, a self-reported history of daily smoking (i.e., ≥1 cigarette per day), a body mass index (BMI) ≥35 kg/m^2^, systolic blood pressure (SBP) ≥140 mmHg, and diastolic blood pressure (DBP) ≥90 mmHg, pre-bronchodilator forced expiratory volume in 1 second (FEV_1_) or pre-bronchodilator forced vital capacity (FVC) < 1 L (i.e., a marker of impaired lung function in our study population), post-bronchodilator FEV_1_/FVC < 70% or excessive erythrocytosis (i.e., hemoglobin ≥19 g/dL in women and ≥21 g/dL in men) [23]. Of those who agreed to participate in this sub-study, we conducted a preliminary evaluation of eligibility criteria. The interview included a discussion of the study objectives, review of eligibility criteria, procedures, associated risks, and benefits, and consent. Enrollees were then invited to schedule an appointment to perform the 6MWT.

### Six-minute walk test

The 6MWT was performed outdoors along a hard flat course measuring 30 m, following standard guidelines [24]. The 6MWT is a self-paced test and participants can choose their own intensity of exercise. It measures functional capacity as the majority of patients do not achieve maximal sustained exercise capacity. Before the test was performed, study staff measured height, weight, and vital signs including pulse oximetry, heart rate, blood pressure, and level of dyspnea and fatigue using the Borg Scale [25]. After the vital signs were recorded, study staff read out loud instructions adapted from the American Thoracic Society [24] to each participant to complete the 6MWT and gave standardized encouragement during the 6MWT to ensure comparability between participants and sites. After the 6MWT was completed, vital signs and Borg scale were repeated immediately.

### Biostatistical methods

The primary outcome was distance walked during the 6MWT (i.e., 6MWD). The primary risk factor was altitude of residence, defined as high altitude for participants in Puno and sea level for participants in Lima. We used *t* tests to compare continuous variables between groups if normally distributed and Mann–Whitney *U* tests if non-normally distributed. We used chi-square or Fisher exact tests whenever appropriate, if categorical variables. We used multiple linear regression to model 6MWD as a function of altitude of residence and adjusted for age, sex, height, resting heart rate, SBP, change in heart rate between the end of 6MWT and baseline, percent body fat, height-adjusted FVC (FVC/height^2^), self-report of walking ≥10 min at least 1 day per week as a proxy for physical activity, number of people per household, and having completed high school. We then developed site-specific reference equations using multiple linear regressions. The lower limit of normal for the 6MWD was defined as the fifth percentile (i.e., $\hat{\mu }-1.64\times \hat{\sigma }$). We conducted statistical analyses in R (http://www.r-project.org).

## Results

### Participant characteristics

Four hundred participants were invited to participate in the study, but 66 (16%) met at least one of the exclusion criteria: one participant was excluded due to age <35 years old; one participant was excluded because of a FEV_1_ <1 L, nine were excluded due to excessive erythrocytosis; six were excluded because they had a BMI >35 kg/m^2^; 15 were excluded due to a post-bronchodilator FEV_1_/FVC <70%; and 37 were excluded based on a baseline SBP ≥140 mmHg or DBP ≥90 mmHg on the day of the test. Of the remaining 334 participants, 168 resided in Lima and 166 in Puno. Age and gender were similar in both groups. Participants in Lima were shorter in height, had a higher body mass index, higher pulse oximetry, and higher SBP but similar DBP, had a significantly lower resting heart rate, and increased their heart rate by more beats per minute during the 6MWT than participants in Puno (Table 1). Both groups were similar in levels of fasting blood glucose, fasting blood cholesterol, and triglycerides; participants in Lima had lower hemoglobin concentration and higher insulin levels. Participants in Lima had lower levels of difficulty breathing and fatigue, self-rated based on the Borg Scale. Participants in Lima were less likely to have completed secondary school than participants in Puno. One hundred of the 168 participants in Lima migrated from a high altitude birthplace, and 4 of 166 in Puno migrated to Puno from a sea level birthplace. All participants have lived in their respective cities where they performed the 6MWT for at least the past 10 years.

**Table 1:** Participant characteristics

	**Lima**	**Puno**	***P***
Sample size	168	166	
Demographics			
Male,%	48%	43%	0.37
Age in years, mean (SD)	54 (11)	54 (10)	0.86
Clinical data, mean (SD)			
Height, cm	154.8 (8.4)	157.5 (9.3)	<0.01
Weight, kg	67.3 (9.9)	66.4 (10.9)	0.47
Body Mass Index, kg/m^2^	28.0 (3.3)	26.7 (3.4)	<0.001
Systolic blood pressure, mmHg	116 (11)	107 (12)	<0.001
Diastolic blood pressure, mmHg	69 (8)	69 (8)	0.78
Pulse oximetry,%	98.4 (1.2)	89.5 (3.0)	<0.001
Resting heart rate, beats/min	66.9 (9.0)	71.6 (9.3)	<0.001
Heart rate at the end of 6WMT, beats/min	72.2 (10.6)	72.0 (10.6)	0.85
Change in heart rate, beats/min	5.3 (6.8)	0.4 (6.2)	<0.001
Pre-bronchodilator FEV_1_, L	2.8 (0.7)	2.9 (0.8)	0.19
Height-adjusted FEV_1_, L/m^2^	1.1 (0.2)	1.1 (0.3)	0.79
Pre-bronchodilator FVC, L	3.6 (0.9)	3.7 (1.0)	0.12
Height-adjusted FVC, L/m^2^	1.5 (0.3)	1.5 (0.3)	0.51
Pre-bronchodilator FEV_1_/FVC%	77.5 (4.9)	76.7 (5.3)	0.14
Difficulty breathing after 6MWT (Borg Scale)	0.3 (0.6)	0.6 (0.9)	<0.001
Fatigue after 6MWT (Borg Scale)	0.5 (0.9)	0.8 (1.0)	<0.001
Laboratory data, mean (SD)			
hsCRP, mg/dL	3.1 (4.0)	2.1 (3.4)	0.02
Insulin in uUnits/mL	10.4 (9.7)	7.5 (4.8)	<0.001
Glucose, mg/dL	91.6 (10.3)	91.1 (19.9)	0.78
Cholesterol, mg/dL	199.8 (38.8)	199.5 (42.1)	0.95
High density lipoprotein cholesterol, mg/dL	41.0 (11.5)	42.3 (12.0)	0.33
Low-density lipoprotein cholesterol, mg/dL	126.3 (35.2)	125.2 (39.6)	0.78
Triglycerides, mg/dL	162.1 (102.0)	160.1 (107.6)	0.86
Hemoglobin, g/dL	13.5 (1.2)	16.9 (1.5)	<0.001
Hemoglobin A1c, mmol/mol	5.6 (0.4)	5.8 (0.4)	<0.001
Socioeconomic status data			
Completed high school,%	64%	83%	<0.001
Percent employed,%	68%	76%	0.13
People per household, mean (SD)	5.5 (2.3)	4.0 (1.9)	<0.001

### Determinants of the 6MWD

**Figure 1:**
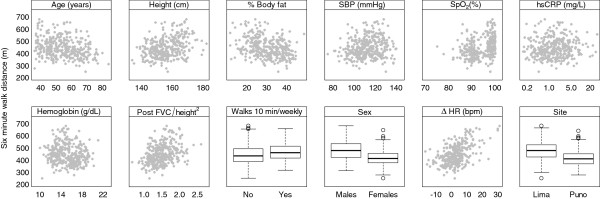
Single variable relationships between 6-min walk distance and selected risk factors.

In single variable analyses, the following variables were important determinants of 6MWD in both the sea level and high altitude groups: sex, age, height, change in heart rate between end of 6MWT and baseline, hemoglobin, lean mass, percent body fat, water weight, education level, weekly exercise, FEV_1_/height^2^, and FVC/height^2^ (Table 2). In Figure 1, we show single variable relationships between 6WMD and selected risk factors. In multivariable linear regression, however, variables that remained important were living at high altitude, age, height, percent body fat, and having completed high school (Table 3). Younger participants walked a greater 6WMD than did older participants with a decrease of 1.4 m per year of older age. Taller participants walked a greater 6MWD than shorter participants with an increase of 1.1 m more per cm increase in height. A greater increase in heart rate from baseline also corresponded to a greater 6MWD, with an increase 4.0 m more per beats/min increase above resting heart rate.

**Table 2:** Predictors of 6MWT

	**Lima**			**Puno**		
	**Number**	**6MWD in m, mean (SD)**	***P***	**Number**	**6MWD in m, mean (SD)**	***P***
Age (years)						
35–44	42	507 (79)	<0.001	32	414 (57)	0.08
45–54	44	494 (58)		60	427 (70)	
55–64	46	469 (86)		44	416 (64)	
65–85	36	421 (72)		30	389 (58)	
Gender (percent)						
Males	80	506 (83)	<0.001	71	446 (68)	<0.001
Females	88	446 (67)		95	392 (51)	
Height (cm)						
130–144	19	431 (60)	<0.001	10	371 (39)	<0.001
145–159	92	450 (73)		86	397 (53)	
160–200	57	529 (70)		70	444 (69)	
Weight (kg)						
40–59	41	440 (76)	<0.001	45	396 (53)	0.06
60–79	107	478 (78)		100	423 (67)	
80–101	20	529 (70)		21	417 (68)	
Body mass index (kg/m^2^)						
16–24	32	473 (89)	0.88	51	428 (77)	0.02
25–29	86	473 (80)		84	417 (57)	
30–35	50	479 (77)		31	387 (54)	
Systolic blood pressure (mmHg)						
75–89	2	448 (106)	0.57	7	399 (36)	0.78
90–119	95	480 (74)		135	415 (64)	
120–139	71	468 (89)		24	419 (74)	
Diastolic blood pressure (mmHg)						
40–64	43	458 (69)	0.24	52	408 (63)	0.64
65–79	107	482 (82)		97	417 (65)	
80–99	18	468 (94)		17	422 (67)	
Resting heart rate (beats per minute)						
40–59	32	465 (67)	0.47	14	438 (67)	0.38
60–79	121	479 (82)		123	413 (64)	
80–105	15	458 (95)		29	412 (65)	
Active heart rate (beats per minute)						
45–64	38	451 (69)	0.05	50	398 (51)	0.08
65–84	113	478 (78)		96	422 (69)	
85–120	17	504 (108)		20	422 (65)	
Delta heart rate (beats per minute)						
< 0	24	447 (49)	<0.001	72	394 (55)	<0.001
0–14	131	470 (79)		92	431 (67)	
15–35	13	569 (87)		2	430 (35)	
Difficulty breathing after 6MWT (self-rating based on Borg Scale)						
0	132	474 (80)	0.65	88	420 (66)	0.39
0.5–2	25	487 (91)		55	413 (63)	
≥ 3	11	462 (64)		23	400 (62)	
Fatigue after 6MWT (self-rating based on Borg Scale)						
0	107	470 (80)	0.41	65	417 (65)	0.80
0.5–2	34	491 (85)		68	416 (63)	
≥ 3	27	474 (76)		33	408 (68)	
hsCRP (mg/dL)						
< 1	50	478 (86)	0.03	76	412 (58)	0.65
1–2	63	492 (81)		63	414 (69)	
3–35	55	452 (70)		27	425 (71)	
Insulin (uUnits/mL)						
0–4	36	479 (90)	0.92	63	427 (76)	0.06
5–10	71	475 (85)		64	400 (48)	
10–100	61	472 (70)		39	419 (65)	
Glucose (mg/dL)						
60–79	17	472 (78)	0.57	30	405 (62)	0.67
80–99	121	478 (83)		110	418 (68)	
100–300	30	461 (71)		26	414 (53)	
High density lipoprotein cholesterol (mg/dL)						
0–39	90	489 (89)	0.04	80	424 (67)	0.21
40–59	66	462 (68)		72	405 (58)	
60–100	12	440 (57)		14	413 (80)	
Triglycerides (mg/dL)						
0–99	52	468 (79)	0.60	37	396 (55)	0.08
100–199	77	474 (78)		92	417 (67)	
200–1,200	39	485 (89)		37	429 (64)	
Hemoglobin (g/dL)						
9–12	58	446 (70)	<0.001	2	416 (39)	<0.01
13–16	110	490 (85)		86	398 (58)	
17–21	0	NA (NA)		78	434 (67)	
Hemoglobin A1c (mmol/mol)						
4–4.99	5	440 (58)	0.37	2	397 (49)	0.22
5–5.99	139	478 (84)		110	415 (67)	
6–7	24	460 (64)		54	416 (60)	
Low-density lipoprotein cholesterol (mg/dL)						
0–74	12	516 (69)	0.21	12	446 (94)	0.22
75–149	118	472 (82)		119	413 (64)	
150–325	38	471 (77)		35	411 (53)	
Lean mass						
30–44	88	442 (70)	<0.001	92	391 (47)	<0.001
45–54	52	496 (73)		43	441 (69)	
55–75	28	536 (79)		31	448 (76)	
Percent body fat (%)						
10–24	40	513 (84)	<0.01	46	455 (74)	<0.001
25–34	79	469 (78)		67	404 (50)	
35–50	49	453 (72)		53	393 (58)	
Water weight (kg)						
20–29	53	438 (69)	<0.001	65	390 (47)	<0.001
30–39	85	475 (74)		69	423 (66)	
40–55	30	540 (78)		32	447 (75)	
Education (level)						
≤ Secondary	60	430 (74)	<0.001	29	382 (42)	<0.01
Higher, non-university	76	489 (72)		52	410 (62)	
University	32	525 (70)		85	429 (68)	
Income per month (soles)						
< 550	27	429 (75)	<0.001	49	399 (63)	0.07
550–1,499	116	483 (77)		69	428 (69)	
≥ 1,500	18	516 (74)		19	410 (52)	
Not available	7			29		
Number of people per household						
1–3	29	465 (99)	0.46	65	416 (71)	0.69
4–5	65	484 (77)		68	410 (59)	
6–17	74	470 (76)		33	422 (66)	
Number of days walking for 10 min or more per week						
0	130	472 (82)	0.34	145	412 (65)	0.17
1	12	508 (82)		5	400 (51)	
≥ 2	26	473 (72)		16	443 (62)	
Number of days of exercise per week						
0	156	470 (79)	0.02	149	410 (62)	<0.01
1	8	550 (67)		5	469 (27)	
≥ 2	4	499 (121)		12	456 (80)	
Pre-bronchodilator forced expiratory volume in 1 s (liters)						
1.0–1.49	4	338 (40)	<0.001	3	346 (30)	<0.01
1.5–1.99	21	414 (64)		18	380 (30)	
*≥* 2.0	143	487 (76)		145	421 (66)	
Height-adjusted pre-bronchodilator forced expiratory volume in 1 s (L/m^2^)						
0.0–0.99	46	429 (76)	<0.001	49	393 (64)	<0.01
1.0–1.49	113	490 (75)		101	420 (64)	
≥ 1.5	9	519 (78)		16	451 (52)	
Pre-bronchodilator forced vital capacity (liters)						
1.0–1.99	2	371 (3)	<0.001	3	346 (30)	<0.001
2.0–2.99	54	429 (67)		40	383 (40)	
≥ 3.0	112	499 (76)		123	427 (67)	
Height-adjusted pre-bronchodilator forced vital capacity (L/m^2^)						
0.0–1.49	94	445 (67)	<0.001	91	395 (58)	<0.001
1.5–1.99	65	510 (79)		63	434 (63)	
≥ 2.0	9	529 (100)		12	464 (71)	
Pre-bronchodilator FEV_1_/FVC (%)						
60–69	11	482 (110)	0.92	20	418 (72)	0.95
70–84	148	474 (80)		138	414 (65)	
≥ 85	9	481 (56)		8	417 (32)	
Pulse oximetry (percent)						
70–84	0	NA (NA)	0.40	8	401 (58)	0.66
85–94	1	406 (NA)		153	415 (65)	
95–100	167	475 (81)		5	434 (72)	

**Table 3:** Multivariable linear regression of 6-min walk distance (6MWD) in meters

	**Change in 6MWD, meters (95% CI)**	***P***
City (Lima is reference)	-47.6 (-81.7 to -13.6)	<0.01
Age (years)	-1.4 (-2.2 to -0.7)	<0.001
Sex (male as reference)	-13.5 (-39.8 to 12.8)	0.32
Height (cm)	1.1 (0.1 to 2.1)	0.04
Percent body fat (%)	-1.4 (-2.6 to -0.3)	0.02
Systolic blood pressure (mmHg)	0.0 (-0.5 to 0.6)	0.87
Pulse oximetry (%)	1.0 (-1.7 to 3.8)	0.45
Log hsCRP (mg/dL)	5.0 (-1.7 to 11.3)	0.12
Hemoglobin (g/dL)	2.4 (-4.5 to 53.3)	0.44
Height-adjusted post-FVC (L/m^2^)	24.4 (-4.1 to 52.9)	0.09
Walks ≥10 min at least 1 day per week	14.6 (-1.4 to 30.6)	0.07
Resting heart rate (beats/min)	0.6 (0.0 to 1.3)	0.07
Change in heart rate between end of 6WMT and baseline (beats/min)	4.0 (3.0 to 4.9)	<0.001
Completed high school	24.9 (10.1 to 39.7)	<0.01
Number of people per household	-1.6 (-4.5 to 1.3)	0.28

### Differences in 6MWD between participants at altitudes of residence

In unadjusted analysis, living at high altitude was an important determinant of 6MWD. Specifically, average 6MWD was 415 m (SD = 65) in Puno vs. 475 m (SD = 81) in Lima (difference of 60 m, 95% CI 44 to 76; *p* < 0.001). In multivariable linear regression, this difference remained such that participants in Puno walked 47.6 m less than did those in Lima (95% CI 13.6 to 81.7 m; *p* = 0.006). There was no difference in 6MWD between those participants in Lima who were born at high altitude vs. born at sea level (*p* = 0.21) and the sample in Puno consisted almost entirely of participants born at high altitude. While we reported a difference in change in heart rate between the end of the 6MWT and baseline in Puno vs. Lima (mean 0.4 vs. 5.3 beats/min, respectively; *p* < 0.001), we did not find a difference in change in pulse oximetry (mean 0.54% vs. 0.41%; *p* = 0.27) or SBP (7.6 mmHg vs. 7.1 mmHg; *p* = 0.70) between the end of the 6MWT and baseline.

### Reference equations for 6MWD in Lima and Puno, Peru

We defined site-specific reference equations for the 6MWD in Lima and Puno, adjusted for age, height, weight, sex, and change in heart rate between the end of test and baseline (Table 4). These models explained 54% and 31% of the variation in 6WMD observed in Lima and Puno, respectively.

**Table 4:** Reference equations for 6-min walk distance in Lima and Puno, Peru

**Lima (*n*** **= 168)**			**Puno (*n*** **= 166)**		
**6MWD (m)**	***R***^**2**^	**Subtract 1.64 ×** ***σ *** **for LLN**	**6MWD (m)**	***R***^**2**^	**Subtract 1.64 ×** ***σ *** **for LLN**
$\begin{array}{ll} 496.8-2.7\times & \left(\mathrm{age}-53.9\right)+1.6\\ & \times \left(\mathrm{height}-156.0\right)+0.9\\ & \times \left(\mathrm{weight}-66.8\right)-38.8\\ & \times I\left(\mathrm{female}\right) \end{array}$	36.2%	106.8	$\begin{array}{ll} 433.0-0.9\times & \left(\mathrm{age}-53.9\right)+2.4\\ & \times \left(\mathrm{height}-156.0\right)-1.3\\ & \times \left(\mathrm{weight}-66.8\right)-37.8\;\\ & \times I\left(\mathrm{female}\right) \end{array}$	24.6%	92.9
$\begin{array}{ll} 468.8-2.4\times & \left(\mathrm{age}-53.9\right)+1.1\\ & \times \left(\mathrm{height}-156.0\right)+0.7\\ & \times \left(\mathrm{weight}-66.8\right)-37.5\\ & \times I\left(\mathrm{female}\right)+5.1\\ & \times \left(\Delta \;\mathrm{Heart}\;\mathrm{rate}\right) \end{array}$	54.1%	90.9	$\begin{array}{ll} 434.3-1.2\times & \left(\mathrm{age}-53.9\right)+2.0\\ & \times \left(\mathrm{height}-156.0\right)-1.3\\ & \times \left(\mathrm{weight}-66.8\right)-40.8\\ & \times I\left(\mathrm{female}\right)+2.68\\ & \times \left(\Delta \mathrm{Heart}\;\mathrm{rate}\right) \end{array}$	30.5%	89.6

Functions of age (in years), height (in cm), weight (in kg), sex, and change in heart rate between the end of 6MWT and baseline (beats/min).

## Discussion

It is well documented that high altitude natives in several settings around the world have made significant adaptations to life in a hypoxic environment [1-9,26,27]. The majority of these studies quantified these adaptations by testing the exercise capacity of highlanders and lowlanders at increasing altitudes and demonstrated highlanders had an overall lower decrease in exercise capacity than lowlanders. Instead of this approach, our study quantified the distance walked during a 6-min walk test by highlanders and lowlanders in their respective environments to determine to what extent high altitude adaptations have allowed high altitude natives to approach the functional capacity of a similar population at sea level. We found that, after controlling for multiple variables, highlanders at their altitude of reference had a lower functional capacity than did lowlanders at sea level, i.e., a 48-m difference in 6-min walk distance. This difference was greater than the minimal clinically important difference for many chronic respiratory conditions (Table 5) [10,28,29].

**Table 5:** Minimal clinical important differences (MCID) of 6-min walk distance (6MWD) for various chronic respiratory diseases

**Condition**	**MCID for 6MWD (m)**
Chronic obstructive pulmonary disease [28]	25 to 35
Idiopathic pulmonary fibrosis [10]	24 to 45
Pulmonary arterial hypertension [29]	33

A shorter 6-min walk distance we observed indicating a 13% lowered functional capacity is comparable to the previously reported decrease in marathon time performed by a trained high altitude native on a course ranging from 4,100 to 4,700 m in elevation. This time was 18.5% slower than the world record at sea level but still faster than the predicted time for a sea level native on a marathon course at this altitude [30]. Despite the superior oxygen uptake and pulmonary gas exchange, a 40% higher diffusing capacity and greater lactic acid buffering in high altitude natives [31,32], there is still a measurable impairment in functional capacity at their altitude of residence. It is unclear, however, whether the decrease in 6-min walk distance is an incomplete adaptation or a protective adaptation among Andean high altitude dwellers. The psychomotor slowing observed in European, Native American, and African altitude groups could be an adaptive rather than a deficient trait, perhaps enabling accuracy of cognitive activity in hypoxic conditions [33]. Hochachka et al. have shown that lower region-by-region brain glucose metabolic rates in high altitude Quechuas compared to lowlanders, which may be the result of a functional adaptation against chronic hypoxia [34]. The lower change in heart rate before and after the 6-min walk test in high altitude vs. sea level dwellers may also help explain the observed shorter 6-min walk distance. Heart rate is affected by both the sympathetic and parasympathetic nervous systems; a balance which is known to be different in hypoxic environments, even after prolonged exposure [35,36]. Some suggest that the lack of increase in heart rate may be a protective functional adaptation which prevents an excess of adrenergic stimulation during exercise [35-37].

The effects of high altitude on human health have been studied using various approaches ranging from its effects on athletic performance [5,6,26], to consequential functional adaptations that arise among highlanders including chronic mountain sickness and high-altitude pulmonary hypertension [38,39]. Maximal cardiopulmonary testing including peak VO_2_ has been shown to predict exercise capacity but has its limitations because the very sick are unable to undergo this testing. In several examples, a primary outcome of functional capacity has been adequately demonstrated using the 6MWT in several instances whereby the study population that was unable to complete other means of maximal cardiopulmonary testing [10-13,15,16,18]. The 6-min walk test is simpler than full cardiopulmonary testing, and in this study, we demonstrate its simplicity in epidemiological studies to compare functional capacity of two healthy populations at different altitudes.

The results of this study suggest a lowered functional capacity among high altitude residents of Puno, Peru, vs. residents at sea level of Lima, Peru using a simple and noninvasive test. The study design does not allow us to conclude causation of the lowered functional capacity among high altitude natives due to its cross-sectional design. This study was also limited to a relatively homogeneous population from two Peruvian cities, which limits external validity. There are also limitations in the study's high altitude population as it includes only decedents of Andean background, which differ in adaptations to hypoxia compared to Tibetan populations [40].

## Conclusions

In summary, after stratifying for variables shown to be associated with the 6-min walk test, there was an important difference in the 6-min walk distance between healthy adult residents at high altitude versus sea level. This 48-m difference shown by multivariable regression is greater than the minimal important difference observed in previous studies for various chronic respiratory diseases, which varies between 24 and 45 m [10,28,29]. Based on the results of this study, measured differences in 6-min walk distance suggests that residents from the city of Puno, Peru, located at high altitude (3,825 m above sea level) have not developed the adaptations to their high altitude environment that are necessary to bring them to an equivalent functional capacity as similar residents of Lima, Peru, located at sea level. Alternatively, the shorter 6-min walk distance in healthy adults residing at high altitude versus sea level could be explained by protective mechanisms in which there is a lower psychomotor activity in favor of increased neuro- and cardioprotection.

## Abbreviations

6MWD: 6-min walk distance; 6MWT: 6-min walk test; BMI: body mass index; DBP: diastolic blood pressure; FEV1: forced expiratory volume in 1 s; FVC: forced vital capacity; SBP: systolic blood pressure; VO2 max: maximal oxygen consumption.

## Competing interests

Sponsor had no role in study design, conduct, analysis, interpretation of results, or manuscript writing. The authors declare that they have no competing interests.

## Authors' contributions

DC, JM, RG, ABO, and WC conceived the original study design. DC, LC, RD, TS, and WC were responsible for conduct of the study. RW, FLV, and VDR provided expert guidance in the interpretation of results. All authors contributed equally to the writing of the manuscript. All authors read and approved the final manuscript.
